# Melatonin Improves Ischemia-Induced Circulation Recovery Impairment in Mice with Streptozotocin-Induced Diabetes by Improving the Endothelial Progenitor Cells Functioning

**DOI:** 10.3390/ijms23179839

**Published:** 2022-08-30

**Authors:** Chin-Sung Kuo, Chi-Yu Chen, Hsin-Lei Huang, Hsiao-Ya Tsai, Ruey-Hsing Chou, Jih-Hua Wei, Po-Hsun Huang, Shing-Jong Lin

**Affiliations:** 1 Division of Endocrinology and Metabolism, Department of Medicine, Taipei Veterans General Hospital, Taipei 112201, Taiwan; 2 Institute of Clinical Medicine, National Yang Ming Chiao Tung University, Taipei 112304, Taiwan; 3 Department of Nursing, College of Nursing, National Taipei University of Nursing and Health Sciences, Taipei 112303, Taiwan; 4 Division of Cardiology, Department of Medicine, Taipei Veterans General Hospital, Taipei 112201, Taiwan; 5 Cardiovascular Research Center, School of Medicine, National Yang Ming Chiao Tung University, Taipei 112304, Taiwan; 6 Division of Cardiology, Department of Internal Medicine, Min-Sheng General Hospital, Taoyuan 330056, Taiwan; 7 Department of Nutrition and Health Sciences, School of Healthcare Management, Kai-Nan University, Taoyuan 338103, Taiwan; 8 Department of Critical Care Medicine, Taipei Veterans General Hospital, Taipei 112201, Taiwan; 9 Department of Medical Research, Taipei Veterans General Hospital, Taipei 112201, Taiwan; 10 Taipei Heart Institute, Taipei Medical University, Taipei 110301, Taiwan; 11 Division of Cardiology, Heart Center, Cheng-Hsin General Hospital, Taipei 112401, Taiwan

**Keywords:** diabetes, endothelial progenitor cell, melatonin, neovascularization

## Abstract

Patients with diabetes mellitus tend to develop ischemia-related complications and have compromised endothelial progenitor cell (EPC) function. Melatonin protects against ischemic injury, possibly via EPC modulation. We investigated whether melatonin pretreatment could restore EPC function impairment and improve circulation recovery in a diabetic critical limb ischemia mouse model. Under 25 mM high-glucose medium in vitro, EPC proliferation, nitric oxide production, tube formation, and endothelial nitric oxide synthase (eNOS) phosphorylation were significantly suppressed. Hyperglycemia promoted EPC senescence and apoptosis as well as increased reactive oxygen species (ROS) production. Melatonin treatment reversed the harmful effects of hyperglycemia on EPC through adenosine monophosphate–activated protein kinase-related mechanisms to increase eNOS phosphorylation and heme oxygenase-1 expression. In an in-vivo study, after a 4-week surgical induction of hindlimb ischemia, mice with streptozotocin (STZ)-induced diabetes showed significant reductions in new vessel formation, tissue reperfusion, and EPC mobilization in ischemic hindlimbs compared to non-diabetic mice. Mice with STZ-induced diabetes that received melatonin treatment (10 mg/kg/day, intraperitoneal) had significantly improved blood perfusion ratios of ischemic to non-ischemic limb, EPC mobilization, and densities of capillaries. In addition, a murine bone marrow transplantation model to support these findings demonstrated that melatonin stimulated bone marrow-originated EPCs to differentiate into vascular endothelial cells in femoral ligation-induced ischemic muscles. In summary, this study suggests that melatonin treatment augments EPC function along with neovascularization in response to ischemia in diabetic mice. We illustrated the protective effects of melatonin on EPC H_2_O_2_ production, senescence, and migration through melatonin receptors and modulating eNOS, AMPK, and HO-1 activities at the cellular level. Thus, melatonin might be used to treat the impairment of EPC mobilization and circulation recuperation in response to ischemic injury caused by chronic hyperglycemia. Additional studies are needed to elucidate the applicability of the results in humans.

## 1. Introduction

Diabetes affects the peripheral vasculature greatly, leading to a high rate of amputation in the absence of trauma [1]. Peripheral arterial disease is difficult to manage in diabetic patients due to poor revascularization outcomes [1,2]. Hyperglycemia is a symptom of diabetes and insulin resistance. Persistent hyperglycemia, along with endothelial dysfunction and impaired new blood vessel formation, plays an essential role in diabetic complications [2,3]. The bone marrow-derived endothelial progenitor cells (EPCs) mobilize into circulation exogenously and endogenously by cytokines and ischemia, respectively [4]. Accumulating evidence suggests that neovascularization results from local endothelial cell proliferation and involves vasculogenesis by EPCs derived from bone marrow [4]. Patients with diabetes have poor EPC function and are more likely to develop ischemic macro- and micro-vascular complications such as nephropathy, accelerated atherosclerosis, retinopathy, and critical limb ischemia [5,6]. Although many studies have been reported to specifically examine the influence of diabetes on angiogenesis in diseases of the peripheral vasculature, limited therapeutic strategies can be provided to improve the outcomes of critical limb ischemia in the context of diabetes.

Melatonin, a pineal hormone, behaves with a circadian endogenous secretion pattern [7]. This pattern is governed by the suprachiasmatic nuclei along with the light cycle; it conveys circadian information for the regulation of physiological functions such as immune system activity, glucose metabolism, and sleep [7]. Recent reviews demonstrate that the impairment of melatonin production is involved in several cardiovascular abnormalities, including hypertension and diabetes [8,9]. Melatonin has receptor-dependent chronobiotic effects, through membrane bound receptors MT1 and MT2 [10], and receptor-independent effects, as an antioxidant that directly scavenges free radicals or indirectly stimulates anti-oxidative enzymes [11,12,13]. Some of the effects could be due to the action of its metabolites [14], since its peripheral metabolism is very rapid through different pathways [15]. It has protective effects against ischemia/reperfusion injury in several organs through the reduction of oxidative damage to the mitochondria [16] and other mechanisms [11,17]. Moreover, melatonin is protective via anti-apoptosis, promoting the secretion of proangiogenic/mitogenic cytokines from mesenchymal stem cells [18] and mitigating ischemia/reperfusion injury [19,20].

Melatonin has been reported to diminish oxidative stress in diabetes [21,22]. It still remains unclear about its therapeutic effect on critical diabetic vascular complications. Thus, this study was performed to investigate whether melatonin improves circulation recovery as well as neovascularization in hindlimb tissues with surgically induced ischemia in a diabetic murine model. Under high-glucose (HG) circumstances in vitro, the effects of melatonin treatment on EPC senescence, viability, and migration were explored.

## 2. Results

### 2.1. Characterization of Human EPCs

Initially, seeded total mononuclear cells (MNCs) from peripheral blood were round. At 4 days, after the medium had been changed, spindle-shaped and elongated early EPCs were observed. Late EPCs were grown to confluence with a cobblestone-like morphological appearance resembling that of mature endothelial cells. The EPCs revealed both hematopoietic stem cells and endothelial surface markers including eNOS, VE-cadherin, KDR, CD34, and CD133 as well as showed simultaneous binding of ulex europaeus lectin (labeled with fluorescein isothiocyanate, green) together with the endocytosed DiI-acLDL (Supplementary Figure S1).

### 2.2. Effects of Melatonin on EPC Function under HG Conditions

**Figure 1:**
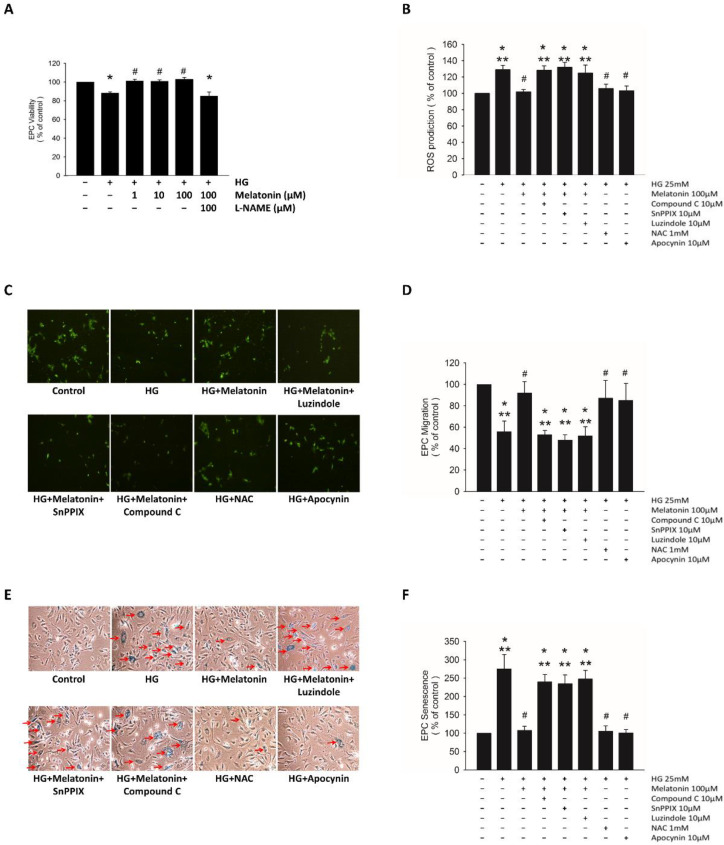
Effects of melatonin on EPC viability, ROS production, and EPC migration and senescence in an HG environment. (**A**) EPC viability, analyzed by Cell Counting Kit-8 assay. (**B**) ROS producing, detected by 2′,7′-dichlorodihydrofluorescein diacetate. (**C**,**D**) EPC migration, examined via modified Boyden chamber. The migrating cells showed green in color under fluorescence microscopic high-power fields. (**E**,**F**) EPC senescence, indicated by the arrows, assessed by acidic-β-galactosidase activity assay. * *p* < 0.05 vs. control, # *p* < 0.05 vs. HG, ** *p* < 0.05 vs. HG + melatonin. Values are shown as means ± standard deviations (3 technical repeats). EPC, endothelial progenitor cell; ROS, reactive oxygen species; HG, high-glucose; SnPPIX, tin-protoporphyrin IX; NAC, N-acetylcysteine.

The incubation of EPCs under the HG condition decreased their viability, and melatonin treatment significantly improved this viability (Figure 1A). This beneficial effect was abolished by L-NAME administration (Figure 1A). The HG condition also markedly increased H_2_O_2_ production and cell senescence as well as decreased EPC migration. These effects were significantly reversed through the melatonin treatment (Figure 1B–F). The luzindole treatment for blockage of melatonin receptors consistently diminished its protective effects. Furthermore, the administration of N-acetylcysteine or apocynin had effects similar to those of melatonin on EPC migration, senescence, and H_2_O_2_ production (Figure 1B–F).

The inhibition of AMPK by compound C suppressed melatonin-improved HG-induced H_2_O_2_ production, but had no effect on melatonin-improved EPC migration or senescence. The administration of SnPPIX abolished the improvement of EPC migration, senescence, and H_2_O_2_ production due to melatonin treatment (Figure 1B–F). These findings indicated that melatonin prevents oxidative stress in EPCs through the melatonin receptor and HO-1 pathway in HG circumstances.

### 2.3. Expression of eNOS, HO-1, and AMPK in EPCs with Melatonin Treatment under HG Conditions

**Figure 2:**
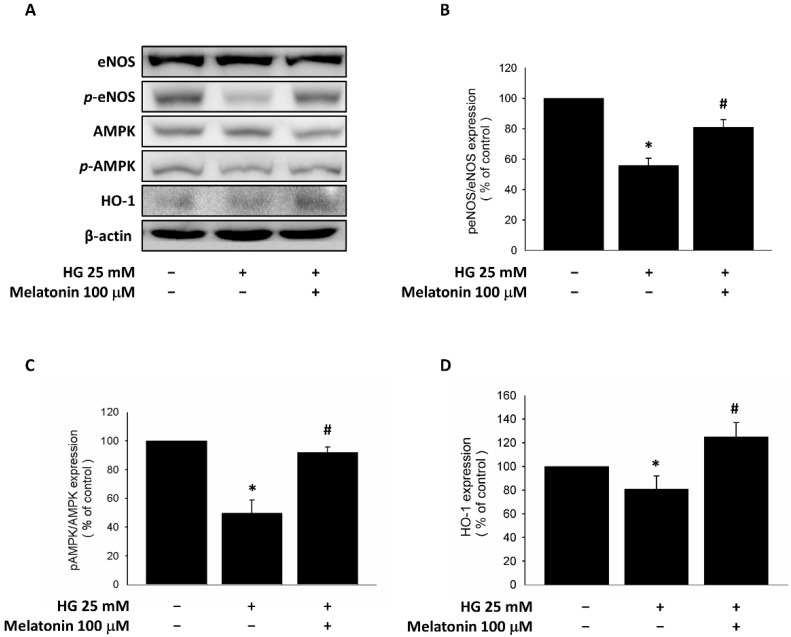
Cellular antioxidant markers of endothelial progenitor cells with melatonin treatment in an HG environment. (**A**) Effects of melatonin on eNOS, AMPK, and HO-1. (**B**) eNOS phosphorylation, (**C**) AMPK phosphorylation, and (**D**) HO-1 expression under HG conditions. * *p* < 0.05 vs. control, # *p* < 0.05 vs. HG. Values are shown as means ± standard deviations (3 technical repeats). HG, high-glucose; eNOS, endothelial nitric oxide synthase; AMPK, adenosine monophosphate–activated protein kinase; HO-1, heme oxygenase-1.

HG-treated EPCs showed significant down-regulation of eNOS phosphorylation at Ser1177, and melatonin treatment upregulated this phosphorylation (Figure 2A,B). These cells also showed suppressed AMPK phosphorylation and HO-1 expression, and melatonin reversed these effects (Figure 2A,C,D). These findings suggest that melatonin improves EPC viability through the eNOS pathway and modulates eNOS, AMPK, and HO-1 activities under HG conditions.

### 2.4. Effects of Melatonin on Ischemia-Induced Circulation Recovery and Circulating EPC Mobilization

**Figure 3:**
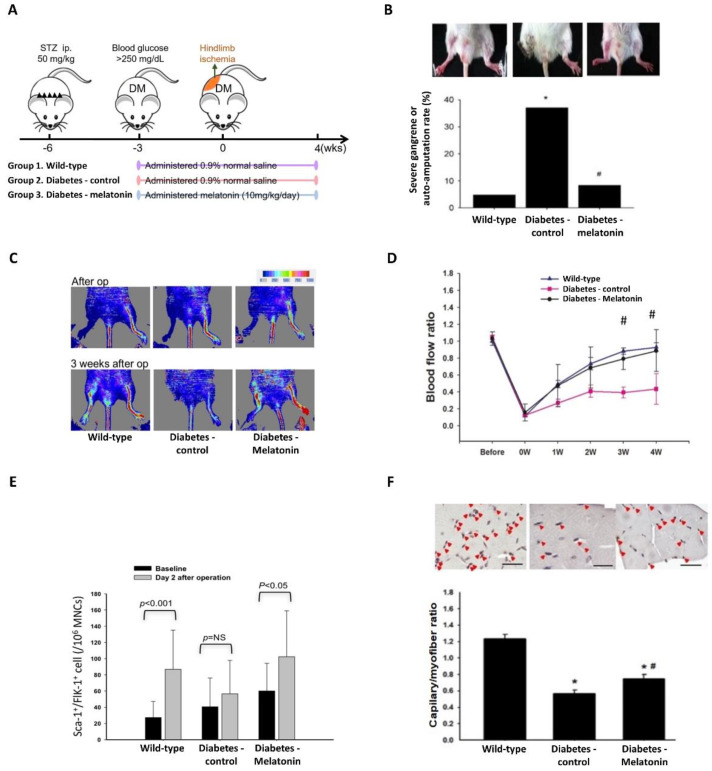
Effects of melatonin on perfusion recovery and neovascularization after ischemic injury. (**A**) Mouse groups and treatment (wild-type, *n* = 21; diabetes-control, *n* = 27; diabetes-melatonin, *n* = 24). (**B**) Vascularity in and loss of ischemic hindlimbs. # *p* < 0.05 vs. diabetes, * *p* < 0.05 vs. wild type, chi-squared test. (**C**) Illustrative pictures of hindlimb perfusion estimated by laser Doppler as well as (**D**) quantification of perfusion recovery, denoted as the ratio of blood flow of the operated side to the contralateral side. (**E**) Before and after femoral ligation, mobilization of endothelial progenitor cell–like (Sca-1^+^/Flk-1^+^) cells. (**F**) Anti-CD31 immunostaining of capillaries. A linear scale, 50 μm. # *p* < 0.05 vs. diabetes, * *p* < 0.05 vs. wild type. Values are shown as means ± standard deviations. Sca-1, stem cell antigen 1; CD-31, cluster of differentiation 31; Flk-1, fetal liver kinase 1.

Some mice had severely impaired vascularity in the ischemic hindlimb, resulting in limb loss after ischemia surgery. Auto-amputation (including that due to severe gangrene) occurred in more STZ-induced diabetic mice (10 of 27 (37%)) than WT mice (1 of 21 (5%)), and treatment with melatonin significantly decreased limb injury (2 of 24 mice (8%); Figure 3B). Laser Doppler imaging revealed delayed circulation recovery after the surgical induction of ischemia in mice with STZ-induced diabetes that did not receive melatonin treatment relative to that observed in WT mice. Mice with STZ-induced diabetes that were treated with melatonin had better blood flow recovery than did those in the diabetes-control group (Figure 3C,D).

Baseline circulating EPC numbers did not differ among WT, STZ-control, and STZ-melatonin mice (Figure 3E). EPC mobilization responding to ischemic injury was observed 2 days after the femoral ligation in WT mice, yet not in STZ-control mice. EPC mobilization was recovered after melatonin treatment in STZ mice (Figure 3E). Our findings suggest that ischemia-related circulation recovery and EPC mobilization were impaired in diabetic mice, and that melatonin treatment recovered these defects.

**Figure 4:**
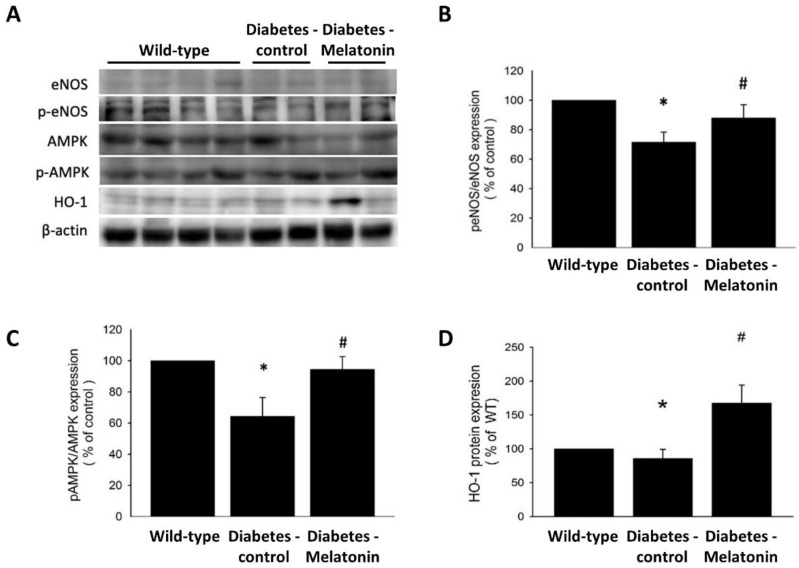
Expression of eNOS, AMPK, and HO-1 protein in ischemic quadriceps. (**A**) In-vivo phosphorylation of eNOS and AMPK, and total protein expressions of (**B**) eNOS, (**C**) AMPK, and (**D**) HO-1. # *p* < 0.05 vs. diabetes, * *p* < 0.05 vs. wild type. Values are shown as means ± standard deviations (wild-type, *n* = 21; diabetes-control, *n* = 27; diabetes-melatonin, *n* = 24, separate experiments in 2 replicates). eNOS, endothelial nitric oxide synthase; HO-1, heme oxygenase-1; AMPK, adenosine monophosphate–activated protein kinase.

In concordance with the findings of laser Doppler analysis, less new vessel formation in mice with diabetes was shown by anti-CD31 immunostaining than in WT mice. In diabetic mice, melatonin treatment increased the density of capillaries detected in the ischemic muscles (Figure 3F). Melatonin administration also enhanced HO-1 protein expression and AMPK and eNOS phosphorylation in ischemic tissues 14 days after ligation surgery (Figure 4A–D). These findings suggest that diabetic mice had attenuated ischemia-induced neovascularization and severe limb damage, and that melatonin treatment reduced the ischemia-induced injury.

### 2.5. EPC Differentiation

**Figure 5:**
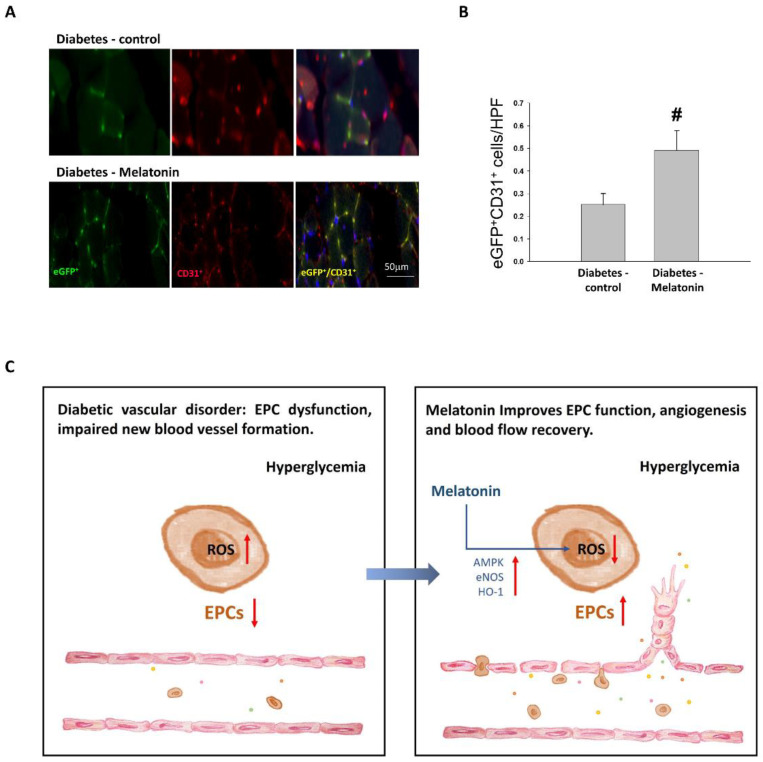
Treatment of melatonin in the bone marrow transplantation model. (**A**) Concomitant expression of enhanced green fluorescent protein (eGFP, marker of transplantation donor) and CD31 (red, endothelial marker) in EPC-derived endothelial cells show yellow in color. A linear scale, 50 μm. (**B**) Density of EPC, calculated by ratios of eGFP^+^/CD31^+^ cell counting in a high-power field (HPF, ×100). The results are shown as means ± standard deviations. # *p* < 0.05 vs. diabetes. (**C**) Melatonin prevented EPC dysfunction and repaired blood flow recovery by increasing the expression of eNOS, AMPK, and HO-1 as well as by lowering oxidative stress. eGFP, enhanced green fluorescent protein; CD31, cluster of differentiation 31, EPC, endothelial progenitor cell.

The melatonin treatment of STZ-induced diabetic mice significantly elevated the differentiation of EPCs derived from bone marrow to endothelial cells compared with the STZ-control in the bone marrow transplantation experiment (Figure 5A–C). This finding supports the observations that melatonin promoted EPC mobilization and enhanced bone marrow cells’ contributions to circulation recovery in ischemia by new vessel formation.

## 3. Discussion

Our findings suggest that daily melatonin administration enhances neovascularization responded to ischemia and EPC function in diabetic mice. First, we found that in-vitro melatonin treatment prevented EPC apoptosis due to HG-induced damage through the eNOS pathway. Second, we identified the protective effects of melatonin on EPC H_2_O_2_ production, senescence, and migration through melatonin receptors and AMPK/eNOS/HO-1 pathways at the cellular level. Third, we showed that melatonin boosted EPC mobilization following the surgical induction of hindlimb ischemia in diabetic mice, suggesting that it enhances EPC function to prevent critical ischemic injury. These findings suggest that melatonin could protect against critical limb damage in diabetes, further advancing knowledge in this field. We confirmed these findings in a murine bone marrow transplantation model, showing that melatonin treatment promoted bone marrow-derived EPCs differentiating into endothelial cells in ischemic quadriceps of STZ mice.

In a recent study, melatonin was found to protect the endothelial functional integrity and its lineage against cellular aging, oxidative stress, and toxicity, as well as to restore perfusion in ischemic limbs, in non-diabetic mice [23]. In addition, daily melatonin administration to human umbilical-vein endothelial cells was found to protect against oxidative stress via upregulation of the SIRT3 pathway [2]. In line with and expanding on these findings, we illustrated that melatonin diminished oxidative stress, migratory dysfunction, senescence, and cell death in EPCs, partly via the AMPK, eNOS, and HO-1 signaling pathway in HG circumstances. These findings of our in vivo studies were consistent with previous findings in non-diabetic mice.

Little is known about how melatonin impacts EPCs, particularly under HG conditions. Jin et al. [24] reported that melatonin fortifies EPCs via stimulation of autophagy flux as well as involving in the AMPK and mTOR pathway. It has also been reported to be vasoprotective through the SIRT3 pathway and has been suggested to decrease ROS [23] and increase nitric oxide [25] bioavailability. In the present study, melatonin protected EPCs from the HG environment via the upregulation of AMPK, eNOS, and HO-1 expression and reduction of ROS. We have explored some critical pathways involved in the enhancement of EPC function under HG conditions via AMPK, eNOS, and HO-1 [26,27,28]. AMPK appears to activate eNOS, given that AMPK activators promote eNOS phosphorylation on Ser1177 [29,30]. Furthermore, the findings of an endothelium-selective constitutively active AMPK transgenic mouse study support the ability of AMPK activation to prevent the diabetes-induced impairment of vascular function by reducing ROS and upregulating HO-1 expression in EPCs and endothelial cells [31].

Patschan et al. [20] reported that melatonin-pretreated EPCs could be used as cell therapy, as they have beneficial effects on kidneys with acute ischemia. Luzindole abolished these effects, implying that they occur via melatonin receptor-mediated pathways. The anti-oxidative effects of melatonin are usually considered to be receptor dependent [11,12,13]. The administration of Luzindole have not been examined in other studies of melatonin and EPCs [23,24,25]. Under hyperglycemia, we found that luzindole abrogated melatonin’s beneficial effects on EPC function.

Melatonin is generally considered safe [32], even up to 1000 mg per day for 1 month in humans [33], or at doses as high as 200 mg/kg/day during gestation in rats [34]. In patients with diabetes, melatonin was also reported to be safe without adverse effects on glucose and lipid metabolism or other routine biochemical tests during long-term use [35]. On the other hand, a few articles found potential side effects of melatonin on glucose homeostasis in melatonin receptor 1B genetic variant subjects [36], possibly on influencing secretion of incretion hormones [37]. Melatonin has been administered by intraperitoneal injection in mice with ischemic injury using daily doses of 4 mg/kg [11], 10 mg/kg [17,24], or 20 mg/kg [23]. We chose the doses of melatonin as 10 mg/kg daily for enhancing EPCs mainly based on the previous report that melatonin 10 mg/kg intraperitoneal injection daily would fortify EPCs [24]. The doses of 10 mg/kg in mice imply equivalent doses of 0.81 mg/kg (around 50 mg/60 kg) in humans [38]. A recent review study for human myocardial ischemia-reperfusion injury found that intravenous route (with doses of 12–49 mg) of administration and early timing regimen benefit the cardioprotective effects of melatonin [39]. Melatonin is commonly administered orally in relatively small doses (1–10 mg) to humans but has a poor and variable bioavailability [40]. Therefore, we injected melatonin intraperitoneally for treatment of the experimental acute ischemic injury. Our findings also tend to support that melatonin is injected early in acute ischemia.

There are some limitations to be mentioned in our study. We examined melatonin injections from 2 weeks before surgery until sacrifice at 4 weeks after surgery in this study. It remains to be elucidated if injections of melatonin at different time points or a different period of injection time lasted will change the outcome. In addition, to minimize the number of experimental mice, we skipped the non-diabetic control + melatonin group which may reveal any potential toxicity of melatonin in mice at the indicated dose (10 mg/kg/day). Moreover, recent published articles showed 20 mg/kg/day melatonin intraperitoneal injection daily without potential toxicity of melatonin in non-diabetic mice [23] and illustrated the effects of different concentrations of melatonin on EPC viability in the absence of high glucose [24]. Furthermore, additional research is needed to clarify the applicability of the study findings in humans as well as the potential side effects. Our study supports future works to explore early melatonin injection with larger doses than common oral doses in ischemic injury.

## 4. Materials and Methods

### 4.1. Cell Culture and Characterization in EPC

We separated MNCs from the peripheral blood of young, healthy volunteers by density gradient centrifugation at 1.077 g/mL (Histopaque-1077, #10771, Sigma-Aldrich, St. Louis, MO, USA) [41,42]. In addition to Endothelial Cell Growth Medium-2 (#CC-4147, Lonza Ltd., Basel, Switzerland) for MNCs seeding at 37 °C and 5% CO_2_ in a six-well plate coated with fibronectin, we added supplements including hydrocortisone, R3-insulin-like growth factor-1, fibroblast growth factor-B, 10% fetal bovine serum, ascorbic acid, epidermal growth factor, gentamicin sulfate–amphotericin-1000, and vascular endothelial growth factor (VEGF). We observed EPC colonization after 2–3 weeks with changing culture medium every 4 days. The EPCs were identified by immunofluorescence. They were labeled with lectins (#L9006, Sigma-Aldrich, St. Louis, MO, USA) and 1,1′-dioctadecyl-3,3,3′,3′-tetramethylindocarbocyanine perchlorate-acetylated low-density lipoprotein (Dil-acLDL, #L3484, Thermo Fisher Scientific, Waltham, MA, USA). Mature endothelial markers were expressed on most of these cells such as CD133 (#130-113-108, Miltenyi Biotec, Bergisch Gladbach, Germany), vascular endothelial (VE)-cadherin (sc-9989, Santa Cruz Biotechnology, Santa Cruz, CA, USA), CD34 (#555821, BD PharMingen, San Diego, CA, USA), kinase insert domain receptor (KDR, #FAB357A, R&D System, Minneapolis, MN, USA), together with endothelial nitric oxide synthase (eNOS, #sc-376751, Santa Cruz Biotechnology, Santa Cruz, CA, USA).

### 4.2. Assessment of EPC Viability

Cells were cultured under HG (25 mM) conditions for 4 days and then treated with 0, 1, 10, or 100 μM melatonin (Sigma-Aldrich, St. Louis, MO, USA) for the examination of their viability by cell counting kit-8 assay (CCK-8, #CK04, Dojindo, Kumamoto, Japan). Melatonin was dissolved in dimethylsulfoxide (DMSO, Sigma-Aldrich, St. Louis, MO, USA) as a 200 mM stock solution and stored at −20 °C [24]. EPCs allocated to a melatonin + eNOS inhibitor group were pretreated with 100 μM Nω-Nitro-D-arginine methyl ester hydrochloride (L-NAME) for 60 min prior to melatonin administration.

### 4.3. Detection of Reactive Oxygen Species Production

Cells were cultured under HG (25 mM) conditions for 4 days and then administered with melatonin (100 μM), compound C (10 μM, an adenosine monophosphate–activated protein kinase (AMPK) inhibitor), SnPPIX (10 μM, a heme oxygenase-1 (HO-1) inhibitor), luzindole (10 μM, a melatonin receptor antagonist), N-acetylcysteine (1 μM, an antioxidant agent), or apocynin (10 μM, an adenine dinucleotide phosphate oxidase inhibitor). Reactive oxygen species (ROS) were assessed by H_2_O_2_ sensing, with 2′,7′-dichlorodihydrofluorescein diacetate (10 μM, 20 min; #D399, Thermo Fisher Scientific, Waltham, MA, USA) serving as the probe by using a microplate reader to examine fluorescence at 485 and 530 nm.

### 4.4. Assessment of EPC Migration

Late EPC migration (crucial for vasculogenesis) was examined using a modified Boyden chamber. Briefly, after the detachment of isolated EPCs using trypsin/EDTA, 4 × 10^4^ EPCs were placed in a 24-well Transwell plate (the upper chamber) with endothelial serum-free growth medium. The lower chamber was placed with medium containing 50 ng/mL VEGF as a chemoattractant across 8-μm pores on a polycarbonate membrane (Transwell, #3428; Corning Inc., Corning, NY, USA). Following incubation for 24 h, we rinsed the membrane quickly using phosphate-buffered saline (PBS) as well as using paraformaldehyde (4%) for fixing. Then, we wiped its upper surface carefully with a cotton ball. The migrating cells were labeled with 1% calcein AM (C1430, Thermo Fisher Scientific) and counted under 6 random fluorescence microscopic high-power fields (HPFs; ×100).

### 4.5. Evaluation of Cellular Senescence in EPC

Cellular senescence was examined with ß-galactosidase served as a label of the acidification that is characteristic of EPC aging. EPCs were washed with PBS and fixed in glutaraldehyde (0.2%) and formaldehyde (2%) for 6 min, subsequently soaked with staining solution (senescence cell staining kit, #CS0030, Sigma-Aldrich; 5 mM potassium ferricyanide, 2 mM MgCl_2_, 1 mg/mL X-gal; pH 6.0) at 37 °C for 12 h in a CO_2_-free environment. We determined the proportion of β-galactosidase-positive cells to total cells counted.

### 4.6. Animals

A mouse model was utilized to examine the impact of melatonin on the neovascularization of diabetic ischemic injury. FVB/NJNarl mice aged 8 weeks obtained from the National Laboratory Animal Centre of Taiwan were randomized into normal (wild-type (WT), *n* = 21), streptozotocin (STZ)-induced diabetes (diabetes-control, *n* = 27), and STZ-induced diabetes + melatonin treatment (diabetes-melatonin, *n* = 24) groups. STZ in citrate solution was injected 40 mg/kg/day intraperitoneally for 5 days to induce experimental diabetes, as described previously [43]. The Institutional Animal Care Committee approved the animal experimental procedures and protocols (IACUC 2015-087 Taipei Veterans General Hospital). We performed the procedures in accord with the Guidelines [44].

### 4.7. Hindlimb Ischemia Surgery

Mice with STZ-induced diabetes were administered vehicle (0.9% normal saline) or melatonin (10 mg/kg) by intraperitoneal injection per day from 2 weeks before surgery until sacrifice at 4 weeks after surgery. Melatonin was dissolved in DMSO at 50 mg/mL and then diluted with normal saline [24]. After 2 weeks of daily melatonin and vehicle administration, the right femoral artery was cut off to induce hindlimb ischemia. By intraperitoneal administration of ketamine (100 mg/kg) together with xylazine (10 mg/kg), we anesthetized the mice. The appropriate depth of anesthesia was maintained by checking that the pinching of the ear, forepaw, and hindpaw with blunt forceps evoked no motor reflex. Ligation of the distal and proximal femoral artery was performed. Laser Doppler analysis (Moor Instruments Limited, Devon, UK) was utilized to assess hindlimb perfusion preoperatively, postoperatively, and weekly thereafter. Ischemic/non-ischemic perfusion ratios were calculated to avoid ambient light/temperature effects.

### 4.8. Flow Cytometry

EPC mobilization was assessed using 100 μL peripheral blood incubated with anti-mouse fluorescein isothiocyanate Sca-1 (#11-5981-82, eBioscience, San Diego, CA, USA) and phycoerythrin Flk-1 (#555308, BD PharMingen, San Diego, CA, USA) by FACSCalibur (Becton-Dickinson, San Jose, CA, USA). Isotype-identical antibodies (#340755, #340013, Becton-Dickinson, Franklin Lakes, NJ, USA) served as controls for the fluorescence-activated cell sorting flow cytometer. After a 30-min incubation, we lysed the cells with PharmLyse (#555899, BD PharMingen, San Diego, CA, USA), then rinsed them with PBS as well as subsequently fixed the cells in paraformaldehyde (2%). Circulating EPCs with Sca-1- and Flk-1-positive (100,000 events each analysis) were analyzed from the centrifugal preparation of MNCs.

### 4.9. Capillary Density Assessment

We euthanized the mice by intraperitoneal administration of ketamine 4 weeks postoperatively. The ischemic limbs were wholly soaked in methanol overnight. Then we embedded the thigh muscles with paraffin. Deparaffinized 5 μm sections were prepared with rat against murine monoclonal antibody CD31 (#550274, BD PharMingen, San Diego, CA, USA). To observe antibody distribution, Vector staining kit (#SK-5100, Vector Laboratories, Burlingame, CA, USA) was applied (red chromogenic substrate and the avidin–biotin complex technique), along with hematoxylin counterstaining. The capillary density (/mm [2]) was obtained by counting visible capillaries (recognized by morphology and CD31 positivity) in 10 randomly chosen areas of each tissue section.

### 4.10. Western Blot Analysis

We lysed EPCs with protein lysis solution (2% sodium dodecyl sulfate (SDS), 10% glycerol, 1 mM phenylmethylsulfonyl fluoride (PMSF), 62.5 mM Tris-HCl, pepstatin, 1 µg/mL aprotinin, and leupeptin). Skeletal muscle tissue lysate was prepared by dissection and homogenized (PRO 200; PROScientific, Oxford, CT, USA) in solution (pH 7.4, 137 mM NaCl, 1 μg/mL pepstatin, 1 mM PMSF, 10 μg/mL aprotinin, 1% NP-40, 5 μg/mL leupeptin 25 mM HEPES). After a 14,000× *g*, 4 °C, 20-min centrifugation and determining supernatant protein concentrations (#5000006, Bio-Rad Laboratories, Inc., Hercules, CA, USA), supernatant specimens (50 μg) were settled by SDS polyacrylamide gel electrophoresis. Immunoblotting was then performed, and protein abundance was determined using antibodies against HO-1 (#sc-136960, Santa Cruz Biotechnology, Santa Cruz, CA, USA), eNOS (#32027, Cell Signaling Technology, Danvers, MA, USA), phosphorylated eNOS (#9571, Cell Signaling Technology, Danvers, MA, USA), AMPK (#5832, Cell Signaling Technology, Danvers, MA, USA), phosphorylated AMPK (#2535, Cell Signaling Technology, Danvers, MA, USA), and β-actin (ab8227, Abcam, Burlingame, CA, USA). Chemiluminescence was used for detection (#NEL105001EA, PerkinElmer Life Science, Boston, MA, USA) along with horseradish peroxidase-conjugated secondary antibodies, and a densitometer was used for protein quantification. β-actin and corresponding proteins were used to normalize proteins and specific protein phosphorylation, respectively.

### 4.11. Bone Marrow Transplantation Model

To demonstrate the impact of melatonin on differentiation of EPC toward endothelial cells and the homing of EPCs in ischemic tissue from bone marrow, we conducted bone marrow transplantation from transgenic-enhanced green fluorescent protein (eGFP; Level Biotechnology Inc., Taipei, Taiwan) mice (FVB background) to WT mice as reported previously [45]. The WT mice (*n* = 8) were lethally irradiated (total dose, 9.0 Gy) at the age of 8 weeks, and were injected with 5 × 10^6^ unfractionated bone marrow cells through tail vein. Two months later, the chimeric mice were intraperitoneally administered STZ for 5 days to induce diabetes and then were divided into diabetes-control (*n* = 4) and diabetes-melatonin (*n* = 4) groups. After 2 weeks of daily melatonin and vehicle administration, the mice received surgery to induce unilateral hindlimb ischemia. Flow cytometry was used to determine that the eGFP-positive bone marrow cell repopulation rate reached 95%. Two weeks postoperatively, tissues were collected for histological and confocal immunofluorescence analyses. Antibodies against GFP (#AB10145, Chemicon, Temecula, CA, USA) and CD31 (#550274, BD PharMingen, San Diego, CA, USA) were used to stain bone marrow-originated EPCs. To estimate the EPC density, eGFP^+^/CD31^+^ (yellow) cells were counted in HPFs (×100) in 6 cross sections from different mice.

### 4.12. Statistical Analysis

All experiments were performed independently at least 3 times, and data were tested for normality using the Shapiro–Wilk test. To compare data between two groups, we utilized unpaired Student’s *t* test. For the comparison of data among multiple groups, we employed one-way analysis of variance with Scheffe post-hoc testing. SPSS (version 23; IBM Corporation, Armonk, NY, USA) software was used for the statistical analysis. Data are shown as means ± standard deviations. *p* values < 0.05 were regarded as statistically significant.

## 5. Conclusions

This study revealed that melatonin is a potent therapeutic agent against experimental hyperglycemia-induced EPC dysfunction, and that it improves the ischemia-induced impairment of circulation recovery and EPC mobilization. The protective effects of melatonin may be through the melatonin receptors and modulating eNOS, AMPK, and HO-1 activities. These findings form a basis for further studies conducted with the aim of developing therapeutic strategies for the vascular complications of diabetes.

## Data Availability

The data that support the findings of this study are available from the corresponding authors upon reasonable request.

## Supplementary Materials

The following supporting information can be downloaded at: https://www.mdpi.com/article/10.3390/ijms23179839/s1.
