# Supplementary material for: Melatonin Improves Ischemia-Induced Circulation Recovery Impairment in Mice with Streptozotocin-Induced Diabetes by Improving the Endothelial Progenitor Cells Functioning
Source: Int J Mol Sci. 2022 Aug 30;23(17):9839. doi: 10.3390/ijms23179839 (PMC9456213; doi:10.3390/ijms23179839)
Supplement: Supplementary file 1 [file ijms-23-09839-s001.zip › ijms-1878513-supplementary.pdf]

Supplementary Materials

Melatonin improves ischemia-induced circulation recovery impairment in mice with streptozotocin-induced diabetes by improving the endothelial progenitor cells functioning

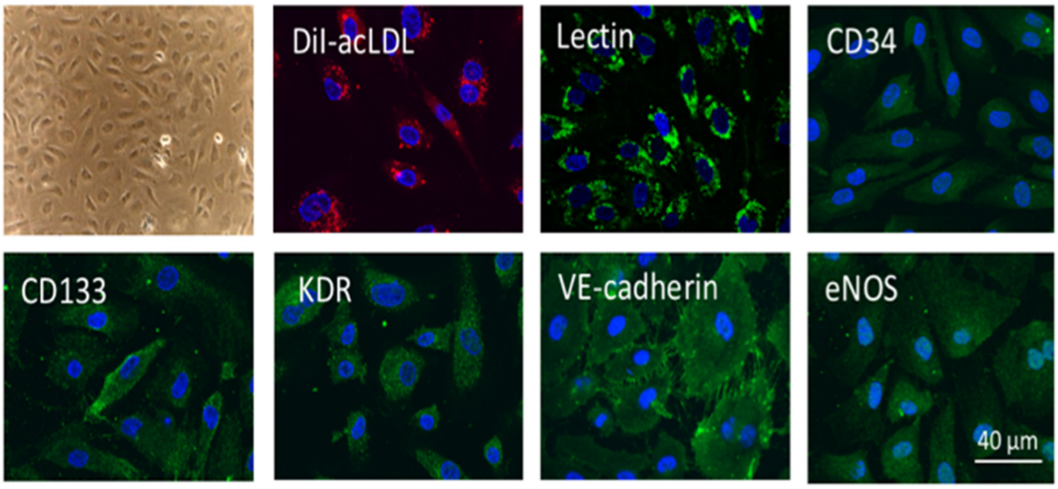

SUPPLEMENTARY FIGURE S1 Characterization of human EPC from peripheral blood. EPC morphology, binding, and expression of endothelial and hematopoietic stem cell surface markers, revealed by nucleus counterstaining with 4′,6-diamidino-2-phenylindole (blue).
